# Micelle encapsulation zinc‐doped copper oxide nanocomposites reverse Olaparib resistance in ovarian cancer by disrupting homologous recombination repair

**DOI:** 10.1002/btm2.10507

**Published:** 2023-03-28

**Authors:** Jingyan Yi, Xin Luo, Jinshan Xing, Aharon Gedanken, Xiukun Lin, Chunxiang Zhang, Gan Qiao

**Affiliations:** ^1^ Department of Medical Cell Biology and Genetics, School of Basic Medical Sciences, Nucleic Acid Medicine of Luzhou Key Laboratory, Key Laboratory of Medical Electrophysiology, Ministry of Education & Medical Electrophysiological Key Laboratory of Sichuan Province, (Collaborative Innovation Center for Prevention of Cardiovascular Diseases), Institute of Cardiovascular Research Southwest Medical University Luzhou Sichuan 646000 China; ^2^ Department of Pharmacology, School of Pharmacy, Nucleic Acid Medicine of Luzhou Key Laboratory Southwest Medical University Luzhou Sichuan 646000 China; ^3^ Department of Neurosurgery The Affiliated Traditional Chinese Medicine Hospital of Southwest Medical University Luzhou Sichuan 646000 China; ^4^ Center for Advanced Materials and Nanotechnology Bar‐Ilan University Ramat Gan 52900 Israel; ^5^ College of Marine Sciences Beibu Gulf University 12 Binhai Road Qinzhou 535011 Guangxi China; ^6^ Nucleic Acid Medicine of Luzhou Key Laboratory, Key Laboratory of Medical Electrophysiology, Ministry of Education & Medical Electrophysiological Key Laboratory of Sichuan Province, (Collaborative Innovation Center for Prevention of Cardiovascular Diseases), Institute of Cardiovascular Research Southwest Medical University Luzhou Sichuan 646000 China; ^7^ School of Pharmacy, Central Nervous System Drug Key Laboratory of Sichuan Province, Nucleic Acid Medicine of Luzhou Key Laboratory, Key Laboratory of Medical Electrophysiology, Ministry of Education & Medical Electrophysiological Key Laboratory of Sichuan Province, (Collaborative Innovation Center for Prevention of Cardiovascular Diseases), Institute of Cardiovascular Research Southwest Medical University Luzhou 646000 Sichuan China

**Keywords:** antitumor mechanisms, HR repair, MEnZn‐CuO NPs, ovarian cancer, PARP inhibitors

## Abstract

Micelle Encapsulation Zinc‐doped copper oxide nanocomposites (MEnZn‐CuO NPs) is a novel doped metal nanomaterial prepared by our group based on Zinc doped copper oxide nanocomposites (Zn‐CuO NPs) using non‐micellar beam. Compared with Zn‐CuO NPs, MEnZn‐CuO NPs have uniform nanoproperties and high stability. In this study, we explored the anticancer effects of MEnZn‐CuO NPs on human ovarian cancer cells. In addition to affecting cell proliferation, migration, apoptosis and autophagy, MEnZn‐CuO NPs have a greater potential for clinical application by inducing HR repair defects in ovarian cancer cells in combination with poly (ADP‐ribose) polymerase inhibitors for lethal effects.

## 1 INTRODUCTION

Ovarian cancer is the most common gynecologic cancer in recent decades, with more than 140,000 deaths worldwide each year.^1^, ^2^ Currently, platinum‐based anticancer drugs dominate the field of chemotherapy for ovarian cancer.^3^, ^4^ However, most relapses in patients are due to dose‐limiting toxicity and the emergence of drug resistance.^5^ In addition, many patients with ovarian cancer have developed advanced disease at the time of diagnosis.^2^ Therefore, there is an urgent need to develop more effective treatments to treat ovarian cancer and delay or prevent recurrence.

The DNA damage response is critical for maintaining genomic stability.^6^, ^7^ When cells suffer DNA damage, they can remove the damage through specific DNA repair pathways, including homologous recombination (HR) repair, non‐homologous end‐joining repair, and single‐strand break repair.^8^ Poly (ADP‐ribose) polymerase (PARP), a DNA damage sensor and signaling sensor can bind damaged DNA at single‐stranded DNA break sites, thereby recruiting DNA repair effectors to the DNA break site.^9^ HR repair is the primary pathway for accurate recovery of DNA double‐strand breaks with high fidelity.^10^ Four PARP inhibitors, Olaparib, Rucaparib, Niraparib, and Talazoparib, have been approved by the FDA for the treatment of recurrent ovarian cancer.^11^ The clinical use of these drugs has favorably altered the outcome of gynecologic malignancies. Approximately 50% of epithelial ovarian cancers exhibit DNA repair damage through HR defects.^12^, ^13^ PARP inhibitors exploit the fundamental weakness of ovarian cancers with HR repair defects and show promising antitumor effects in ovarian cancers with BRCA1/2 mutations.^14^, ^15^, ^16^, ^17^ There is growing evidence that PARP inhibitors are equally efficacious in ovarian cancers without BRCA1/2 mutations, which may be caused by other molecular defects.^17^ PARP inhibitors are an exciting and promising new class of anticancer agents, however, acquired resistance remains a significant clinical hurdle for PARP inhibitors.^11^ Emerging combination therapeutic strategies designed to selectively disrupt HR repair in cancer cells and make them vulnerable to PARP inhibitors have been evaluated in preclinical and early clinical trials in a variety of cancer types, including ovarian cancer.

Compared to conventional therapies, nanocomposites (NPs) offer new opportunities for the development of diagnostic and therapeutic tools for cancer and other diseases, including the possibility of destroying cancerous tumors with minimal damage to normal cells, and the possibility of detecting and destroying cancer cells before they form.^18^, ^19^, ^20^ The physical, chemical, and biological properties of NPs are fundamentally different from their corresponding bulk materials because the quantum mechanical nature of atomic interactions is influenced by their size.^21^ Nanometal oxides (nMeOs) are among the most promising NPs due to their potential physicochemical properties such as high affinity, low molecular weight, and large specific surface area.^22^ nMeOs such as ZnO, CuO, and Fe3O4 NPs have been reported to cause genotoxicity, mitochondrial dysfunction and induction of apoptosis and autophagy in many cancer cell lines.^21^, ^23^, ^24^ The dope NPs is a kind of mechanism that metal ions are doped in the unit cell of the monoclinic lattice replacing some of the metal ions by sono‐chemical method by our collaborator.^25^ Enhanced activity of doped nMeOs has been reported due to their increased structural defects and increased ROS production.^26^, ^27^, ^28^ A zinc‐doped CuO nanocomposite (Zn‐CuO NPs) with efficient antibacterial activity was synthesized by the acoustic chemistry method in our collaborator group previously.^29^ Previous studies have shown that Zn‐CuO NPs can induce apoptosis through ROS‐mediated pathway, thus inhibiting cancer cell proliferation.^30^, ^31^ We also found that Zn‐CuO NPs can have antiglioma effects both in vitro and in vivo.^32^ However, due to their special structure, Zn‐CuO NPs suffer from low solubility. Polymeric micelles as a novel drug delivery system have the advantages of prolonging drug circulation time, enhancing drug accumulation, improving drug dissolution range, and reducing side effects.^33^ In this study, we used surfactants and polyether polyol as stabilizers to prepare stabilized nanoparticles by strong ultrasound technique encapsulation to enhance the durability of metal nanoparticles, reduce their accumulation, enhance their distribution, and form a nanosystem of 50‐nm metal nanomicelles. We continued the characterization using transmission electron microscopy (TEM) and found that the metal nanoparticles were dispersed in the interior of the micellar system and the micellar encapsulation improved the homogeneity of the metal nanoparticles. We named them as Micelle Encapsulation Zinc‐doped copper oxide nanocomposites (MEnZn‐CuO NPs). The results suggest that MEnZn‐CuO NPs increase the range of organic solvent selection, reduce the adverse effects of surfactants, and are more stable in nature. However, it is not clear whether MEnZn‐CuO NPs have greater antitumor potential than Zn‐CuO NPs.

In this study, we examined the therapeutic effects of MEnZn‐CuO NPs in ovarian cancer in vitro and in vivo, and explored the underlying molecular mechanisms. We found that MEnZn‐CuO NPs exerted strong antitumor effects by causing cellular genomic damage. More importantly, MEnZn‐CuO NPs significantly increased the therapeutic sensitivity of ovarian cancer cells to the PARP inhibitor Olaparib by inhibiting the cellular HR repair ability. These findings provide strong evidence that MEnZn‐CuO NPs may be applied as a novel antitumor nanodrug for ovarian cancer treatment, especially for Olaparib‐resistant patients.

## 2 RESULTS

### 2.1 Compared with Zn‐CuO NPs, MEnZn‐CuO NPs have stronger homogeneity and stability

**FIGURE 1:**
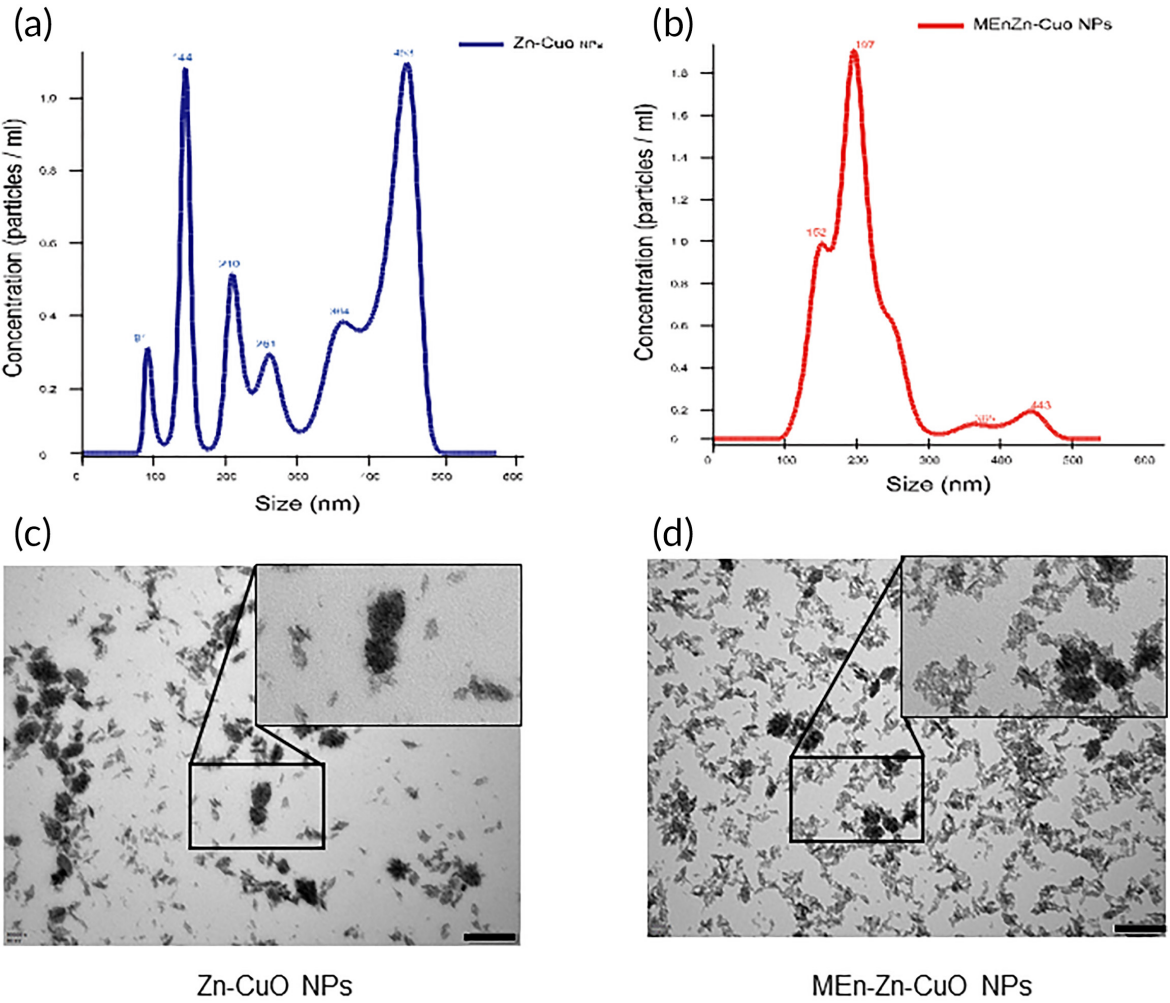
Characterization of MEn‐Zn‐CuO NPs and Zn‐CuO NPs. (a) Size distribution profile of the of MEnZn‐CuO NPs and (b) Zn‐CuO NPs was analyzed by Nanoparticle tracking analysis NanoSight 300 (Malvern Panalytical, Malvern, UK). Images of MEnZn‐CuO NPs (c) and Zn‐CuO NPs (d) at 1 mg/mL and a magnification of MEnZn‐CuO NPs (in the black rectangle) and Zn‐CuO NPs (in the red rectangle) with optimized contrast after applying a Gaussian blurr effect with ImageJ to increase the visibility of the corona layer was identified by high resolution transmission electron microscope (TEM). Scale bar: 200 nm.

Previously, our team reported the anti‐tumor activity of metal nanoparticles Zn‐CuO NPs,^31^ but due to the properties of metal nanoparticles are prone to aggregation as well as liquid settling.^25^, ^30^, ^31^ Therefore, this study proposes to reduce the aggregation between metal nanoparticles and increase the stability of metal nanoparticles in solution by colloidal technique. The particle size distribution of Zn‐CuO NPs was wider, ranging from 100 to 500 nm (Figure 1a), and its particle diameter is mainly distributed at 144 and 453 nm, in addition, it is more distributed at 453 nm. We prepared MEnZn‐CuO NPs by using high‐intensity ultrasound, and the particle size distribution of MEnZn‐CuO was more homogeneous than that of Zn‐CuO NPs, with the particle size around 200 nm (Figure 1b). The TEM results also suggested that Zn‐CuO NPs tend to aggregate into larger metal nanoparticle aggregates compared to MEnZn‐CuO NPs, causing the accumulation of nanoparticles resulting in weakened nanoparticle properties (Figure 1c,d). Figure S1A shown that the micellization could reduce the aggregation of Zn‐CuO by comparing the size of sediment in several days. The above results suggested that our non‐micellar prepared Zn‐CuO NPs are not stable enough in liquid, while MEnZn‐CuO NPs, after micellization treatment, are less likely to aggregate and have more homogeneous nanoscale properties.

### 2.2 MEnZn‐CuO NPs can specifically inhibit the proliferation of ovarian cancer cell lines

**FIGURE 2:**
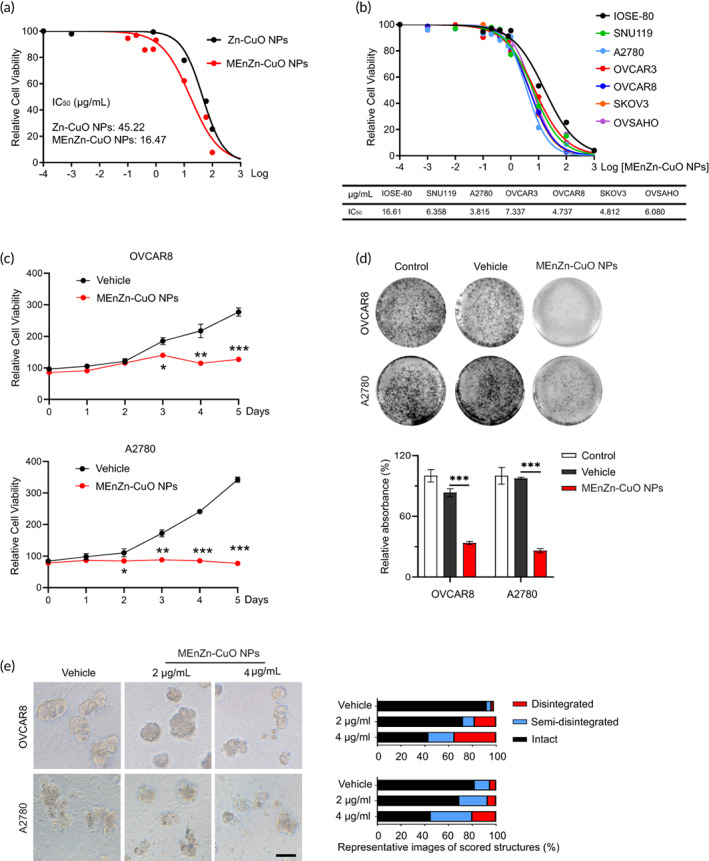
Effects of MEnZn‐CuO NPs on the growth of ovarian cancer cell lines in vitro. (a) IC_50_ curves of two Nano‐particles in the IOSE80 cell. (b) Dose‐response curves of MEnZn‐CuO NPs in a panel of seven cell lines treated with varying concentrations for 72 h. (c) Time inhibitory effect of the MEnZn‐CuO NPs on the ovarian cancer cell lines OVCAR8 and A2780. (d) The MEnZn‐CuO NPs inhibited the colony formation in ovarian cancer cells, significantly. (e) Ovarian cancer cell lines were cultured in 3D matrigel and MEnZn‐CuO NPs‐treated for 10–15 days. Error bars represent standard deviations (SD) from the mean. Representative images of cells are shown. Quantification of scored structures (intact, semi‐disintegrated and disintegrated) is shown. Scale bar, 50 μm. *p < 0.05; **p < 0.01; ***p < 0.001 (Student's *t*‐test).

To compare the drug potency of Zn‐CuO NPs and MEnZn‐CuO NPs, we assayed the IC_50_ of both Zn‐CuO NPs and MEnZn‐CuO NPs in normal ovarian epithelial cells IOSE80. the drug sensitivity of MEnZn‐CuO NPs was increased nearly 3‐fold in IOSE80 compared with Zn‐CuO NPs (Figure 2a). We next evaluated the inhibitory effect of MEnZn‐CuO NPs in a group of ovarian cancer cell lines. After 72 h of drug action, cytotoxic effects were determined by the CCK‐8 assay, followed by a median‐median IC50s analysis. All six ovarian cancer cell lines were more susceptible to MEnZn‐CuO NPs compared to IOSE80, with A2780 and OVCAR8 being the most prominent (Figure 2b). Because the ZnCuO could inhibited the cell growth by the autophagy enhancing, the autophagy phenotype was test by the double label autophagy. As shown in the Figure S1C, the cell lines A2780 and OVCAR8 was treatment with the Zn‐CuO NPs (4 μg/mL) and MEnZn‐CuO NPs (4 μg/mL), the MEnZn‐CuO NPs shown more fluorescent intensity of red than the control group and Zn‐CuO NPs treatment group. MEnZn‐CuO NPs have more autophagy transmission capacity than the Zn‐CuO NPs. So, the MEnZn‐CuO NPs could be an autophagy enhancer to inhibited the cells growth. We further examined the drug responses of A2780 and OVCAR8 to MEnZn‐CuO NPs over a long period, and the results showed that the metal nanoparticles produced greater inhibition of proliferation of both cell types (Figure 2c,d). To evaluate the role of drugs in tumor formation and progression in vivo under conditions that more closely mimic the tumor microenvironment, we cultured two ovarian cancer cell lines as three‐dimensional (3D) spheres in Matrigel. Both concentrations of MEnZn‐CuO NPs induced some degree of disintegration of the cell spheres, and a greater degree of structural disintegration occurred in the two ovarian cancer cell lines upon treatment with higher concentrations of the drug (Figure 2e). Taken together, these data suggest that MEnZn‐CuO NPs have great potential for significantly inhibitory effects on ovarian cancer cell lines.

### 2.3 MEnZn‐CuO NPs significantly inhibited the migration ability of ovarian cancer cell lines and increased their apoptosis and autophagy

**FIGURE 3:**
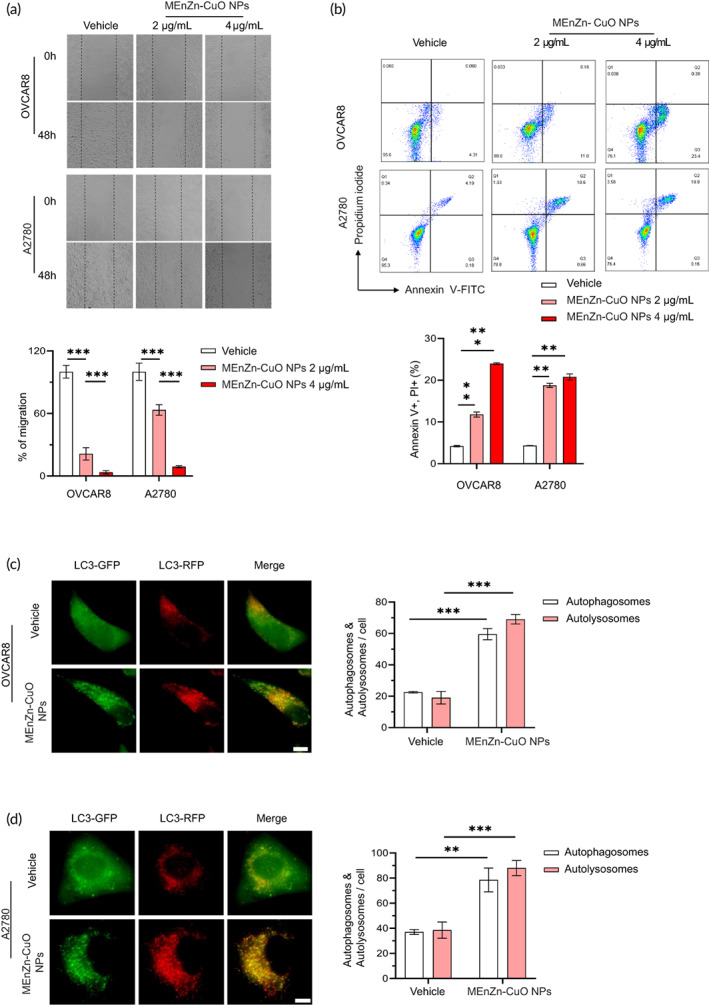
MEnZn‐CuO NPs significantly affected the migration, apoptosis and autophagy of OVCAR8 and A2780 ovarian cancer cell lines. (a) MEnZn‐CuO NPs inhibited the cell migration by scratch wound healing. (b) The apoptosis of OVCAR8 and A2780 ovarian cancer cell lines treated with MEnZn‐CuO NPs was identified by flow cytometry analysis. The autophagy of OVCAR8 (c) and A2780 (d) ovarian cancer cell lines treated with MEnZn‐CuO NPs was analysis by fluorescence microscope. Red puncta represent autolysosomes and yellow puncta represent autophagosomes *p < 0.05; **p < 0.01; ***p < 0.001 (Student's *t* test).

To further investigate the effect of MEnZn‐CuO NPs in ovarian cancer cell lines, we used a series of phenotypic experiments for evaluation. We first examined the effect of MEnZn‐CuO NPs on the migration ability of two ovarian cancer cell lines (A2780 and OVCAR8). The results showed that MEnZn‐CuO NPs significantly downregulated the expression of MMP2 and MMP9, which are indicators of cell migration, and the phenotypic assays also suggested that the drug significantly inhibited the migration ability of both ovarian cancer cell lines (Figure 3a and S1D). Next, we proceeded to evaluate the effect of MEnZn‐CuO NPs on the apoptosis of ovarian cancer cell lines. The upregulation of Cleaved‐parp and enrichment of Annexin V/PI positive cells indicated that A2780 and OVCAR8 cell lines underwent significant apoptosis under MEnZn‐CuO NPs treatment (Figure 3b and S1). Previous studies have shown that Zn‐CuO NPs can induce the production of autophagy in tumor cells. Therefore, we examined the effect of MEnZn‐CuO NPs on autophagy in ovarian cancer cell lines. The autophagic flux assay confirmed that MEnZn‐CuO NPs could also induce autophagy in cells. As shown in Figure S1B, there were no differences in retroviral transfection efficiency with 80% positive cells before the nanoparticle treatment. As shown in Figure 3c,d, mGFP‐RFP‐LC3 retroviral transfection of ovarian cancer indicated changes in autophagy with significantly more red spots in the cells after MEnZn‐CuO NPs. mCherry‐GFP‐LC3B fusion protein does not fuse with lysosomes if green fluorescent light and red fluorescent light co‐localize in cells. When red fluorescence was enhanced without green fluorescence, it indicates that mCherry‐GFP‐LC3B fusion protein was localized in lysosomes or autophagolysosome, which mean that autophagic flux has been activated. Meaning that MEnZn‐CuO NPs promoted to the autophagic flux with the production of autophagosomes. In addition, MEnZn‐CuO NPs also showed significant upregulation of ATG7 and p‐ULK after treatment of two ovarian cancer cell lines (Figure S1). The above data suggest that MEnZn‐CuO NPs can significantly inhibit the migration ability of ovarian cancer cell lines and increase the level of apoptosis and autophagy in tumor cells.

### 2.4 MEnZn‐CuO NPs can cause defective HR repair ability in ovarian cancer cell lines

**FIGURE 4:**
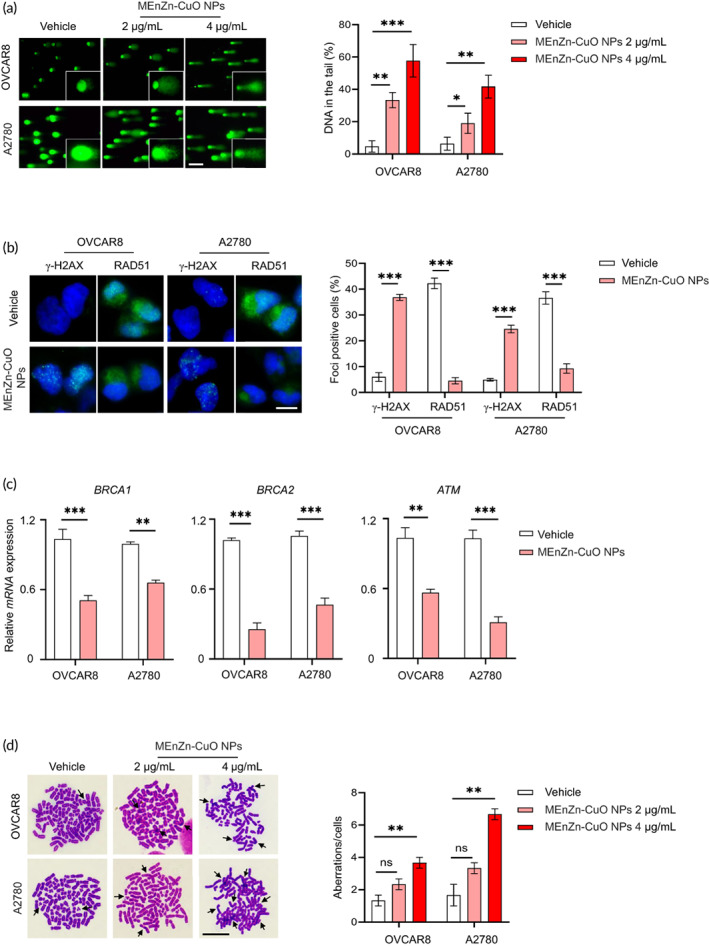
MEnZn‐CuO NPs induced DNA damage and chromosome instability in ovarian cancer cell lines. (a) DNA damage in A2780 and OVCAR8 ovarian cancer cell lines after 48 h treatment with MEnZn‐CuO NPs was examined by comet assay. Scale bar, 100 μm. (b) The expression of γH2AX and RAD51 in A2780 and OVCAR8 ovarian cancer cell lines after 48 h treatment with MEnZn‐CuO NPs was detected by immunofluorescence staining. Scale bar, 20 μm. Cells containing more than five foci were scored as positive. (c) The mRNA level of HR gene BRCA1, BRCA1and ATM in A2780 and OVCAR8 ovarian cancer cell lines treated with MEnZn‐CuO NPs as indicated for 24 h was identified by quantitative transcription PCR analysis. (d) The chromosome aberrations in A2780 and OVCAR8 ovarian cancer cell lines after 48 h treatment with MEnZn‐CuO NPs was detected by metaphase chromosome spread assay. Scale bar, 10 μm. Mean ± S.D. for three independent experiments were shown. *p < 0.05; **p < 0.01; ***p < 0.001 (Student's *t* test).

Both A2780 and OVCAR8 are BRCA wild‐type ovarian cancer cell lines and therefore have a relatively well‐developed HR repair system. This prompted us to investigate whether MEnZn‐CuO NPs affect the HR repair capacity of ovarian cancer cell lines. To this end, we evaluated DNA damage in A2780 and OVCAR8 cell lines after treatment with MEnZn‐CuO NPs. Comet assay showed that low concentrations of MEnZn‐CuO NPs induced DNA damage compared to vehicle, and we also noted that high concentrations of drug treatment led to further accumulation of damaged DNA in the cells (Figure 4a). The next immunofluorescence staining analysis showed that RAD51 nuclear foci (markers of HR repair capacity) were reduced and γH2AX nuclear foci (markers of DNA double‐strand breaks) were increased in two ovarian cancer cell lines (A2780 and OVCAR8) after treatment with MEnZn‐CuO NPs compared with controls (Figure 4b). These results suggest that MEnZn‐CuO NPs may cause defective cellular HR repair capacity. Next, we examined the changes of BRCA1, BRCA2, ATM, and RAD51, key genes of HR repair pathway, in MEnZn‐CuO NPs‐treated ovarian cancer cells. The results showed that all these genes were differentially down‐regulated (Figure 4c and Figure S2). We also used a metaphase chromosome spread assay to assess the effect of MEnZn‐CuO NPs on the genomic integrity of ovarian cancer cell lines. MEnZn‐CuO NPs significantly induced more abnormal chromosome structures compared to the vehicle (Figure 4d), suggesting cellular genomic instability. PARP inhibitors are well‐established and popular drug targets for ovarian cancer treatment, with the important rationale of causing cells to undergo DNA damage, but this often triggers the activation of cellular repair pathways.^34^ Our experimental results prompted us to speculate whether the defective cellular HR repair capacity induced by MEnZn‐CuO NPs would enhance the sensitivity of ovarian cancer cell lines to PARP inhibitors in a synthetic lethal manner.

### 2.5 MEnZn‐CuO NPs and PARP inhibitor Olaparib synergistically inhibit the growth of ovarian cancer cell lines in vitro

**FIGURE 5:**
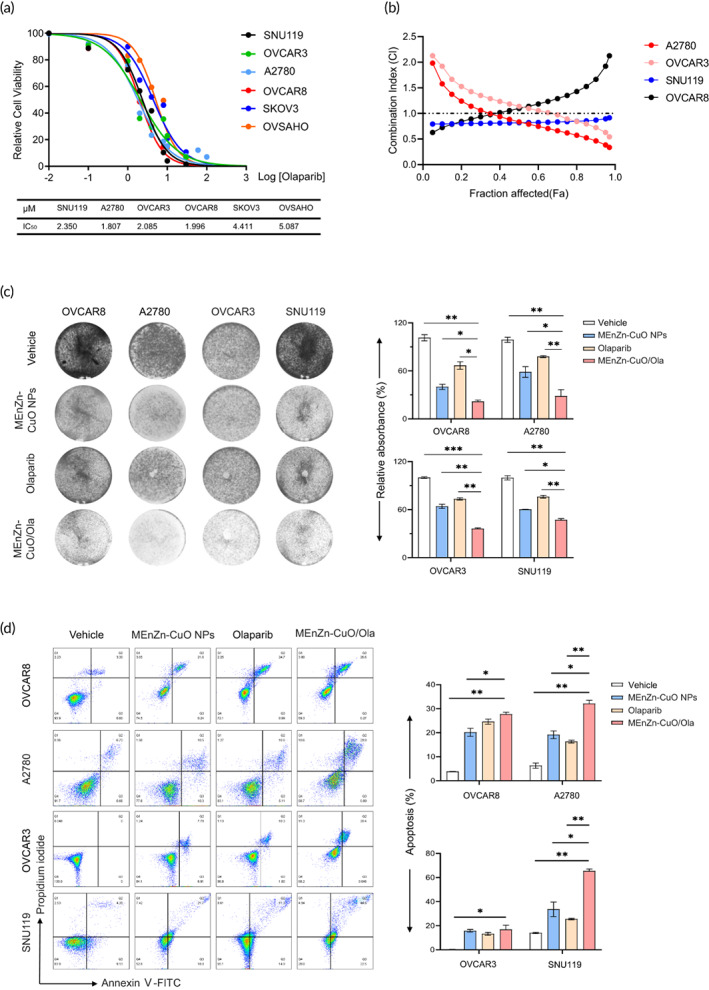
Effects of Olaparib and MEnZn‐CuO NPs monotherapy or in combination on the growth of ovarian cancer cell lines in vitro. (a) Dose‐response curves of Olaparib in a panel of six ovarian cancer cell lines treated with varying concentrations for 72 h. (b) The synergistic effect of Olaparib combined with MEnZn‐CuO NPs in A2780, OVCAR3, OVCAR8, and SNU119 ovarian cancer cells was detected by FA‐CI method. (c) Effect of Olaparib and MEnZn‐CuO NPs alone or in combination on colony formation in A2780, OVCAR3, OVCAR8, and SNU119 ovarian cancer cell lines. Representative images are shown. (d) Flow cytometry analysis of Annexin V and PI‐stained cells to assess the effect of Olaparib and MEnZn‐CuO NPs monotherapy or combination on apoptosis in A2780, OVCAR3, OVCAR8, and SNU119 ovarian cancer cell lines. Mean ± S.D. for three independent experiments were shown. *p < 0.05; **p < 0.01; ***p < 0.001 (Student's *t* test).

To test the conjecture, we first determined the IC_50_ of the PARP inhibitor Olaparib in a set of ovarian cancer cell lines to determine the next concentration to be used (Figure 5a). Next, we examined the susceptibility of ovarian cancer cell lines to the combination of MEnZn‐CuO NPs and Olaparib. CalcuSyn model was used to assess the effect of the combination and four ovarian cancer cell lines (A2780, OVCAR3, SNU119, and OVCAR8) showed a combination effect (Figure 5b and Figure S3A). We further investigated the synergistic growth inhibition of these cell lines by combination treatment through clone formation and 3D culture experiments. Comparing MEnZn‐CuO NPs to Olaparib alone, MEnZn‐CuO NPs and Olaparib displayed significant growth inhibition on A2780, OVCAR3, SNU119, and OVCAR8 cells (Figure 5c and S3B). Hence, the combination of MEnZn‐CuO NPs and PARP inhibitors has synergistic effects against ovarian cancer cell lines. Consistent with the drug‐induced therapeutic effect, the combination of MEnZn‐CuO NPs and Olaparib induced a large number of apoptotic cells compared to the vehicle and single agent, as measured by Annexin V/PI assay (Figure 5d). These data provide further evidence for the synergistic effect of MEnZn‐CuO NPs and PARP inhibitors in ovarian cancer cells.

### 2.6 MEnZn‐CuO NPs induce DNA damage and genomic instability in ovarian cancer cell lines by impairing HR repair capacity in synergy with Olaparib

**FIGURE 6:**
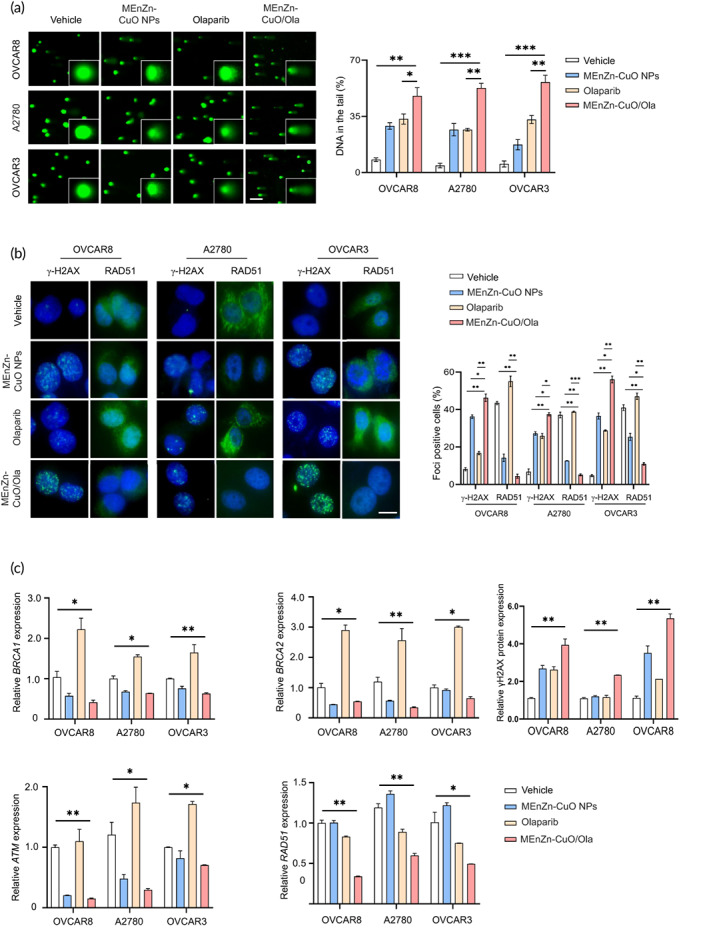
Olaparib in combination with MEnZn‐CuO NPs caused DNA damage and chromosomal instability in ovarian cancer cell lines. (a) DNA damage in A2780, OVCAR8 and OVCAR3 ovarian cancer cell lines was detected by comet assay after 48 h of drug treatment. Scale bar, 100 μm. (b) Expression of γH2AX and RAD51 in A2780, OVCAR8 and OVCAR3 ovarian cancer cell lines was detected by immunofluorescence assay 48 h after drug treatment. Scale bar, 20 μm (c) The mRNA levels of homologous recombinant genes BRCA1, BRCA1 and ATM in A2780, OVCAR8 and OVCAR3 ovarian cancer cell lines were detected by real‐time quantitative PCR assay 24 h after drug treatment. And the quality of relative expression of γH2AX. Mean ± S.D. for three independent experiments were shown. *p < 0.05; **p < 0.01; ***p < 0.001 (Student's *t* test).

Through a previous exploration of the mechanism of drug action of MEnZn‐CuO NPs in ovarian cancer cell lines, we speculated that the synergistic activity of MEnZn‐CuO NPs and PARP inhibitors might be related to HR repair damage. To this end, we evaluated DNA damage in four ovarian cancer cell lines after the combination of the two drugs. The combination of MEnZn‐CuO NPs and Olaparib significantly caused more severe cell trailing compared to the vehicle and single agent groups, suggesting an exacerbation of DNA damage (Figure 6a). Consistent with this, the combination dosing resulted in a significant aggregation of intracellular γH2AX into the nucleus and was accompanied by a reduction in RAD51 nuclear foci (Figure 6b). Metaphase chromosome spread assays similarly demonstrated that MEnZn‐CuO NPs enhanced the effect of Olaparib on the genomic integrity of ovarian cancer cell lines (Figure S4A). After clarifying that the combined effect of MEnZn‐CuO NPs and Olaparib could affect the phenotype of cellular genomic stability, we further examined the changes in key genes of HR repair pathway and DNA damage genes. We found that the levels of BRCA1, BRCA2, ATM, and RAD51 had a significant decrease and γH2AX had a significant increase in the combination group compared to the vehicle, indicating that intracellular HR repair pathway damage remained after the combination (Figure 6c and Figure S4B). To identify the synergy mechanism, the combination of rapamycin (autophagy enhancer), BRCA1‐IN‐2 (inhibitor of HR repair gene BRCA1) with Olaparib were used to treat the OVCAR3 (HR deficient) and A2780 (HR proficient) cell lines. The combination of BRCA1‐IN‐2 with Olaparib (pink curve) resulted in a curve‐shift to the left compared with the BRCA1‐IN‐2 (blue curve) or the Olaparib (purple curve) in the A2780 (Figure S4C). But the curve‐shift was not shown in the combination treatment in the OVCAR3 (Figure S4D). In the combination rapamycin with Olaparib was shown the curve‐shift in the both A2780 and OVCAR3 (Figure S4E and 4F). So that the MEnZn‐CuO NPs may synergy with the Olaparib to inhibited the ovarian cancer by the multiple path, such as the autophagy and chromosome instability, in the HRD or HRR cell lines. These results, along with synergistic cytotoxicity data (Figure 5), are consistent with the hypothesis that MEnZn‐CuO NPs synergy with PARP inhibitor to inhibit the ovarian cancer.

### 2.7 The combined lethal effect of MEnZn‐CuO NPs and Olaparib was effective in vivo

**FIGURE 7:**
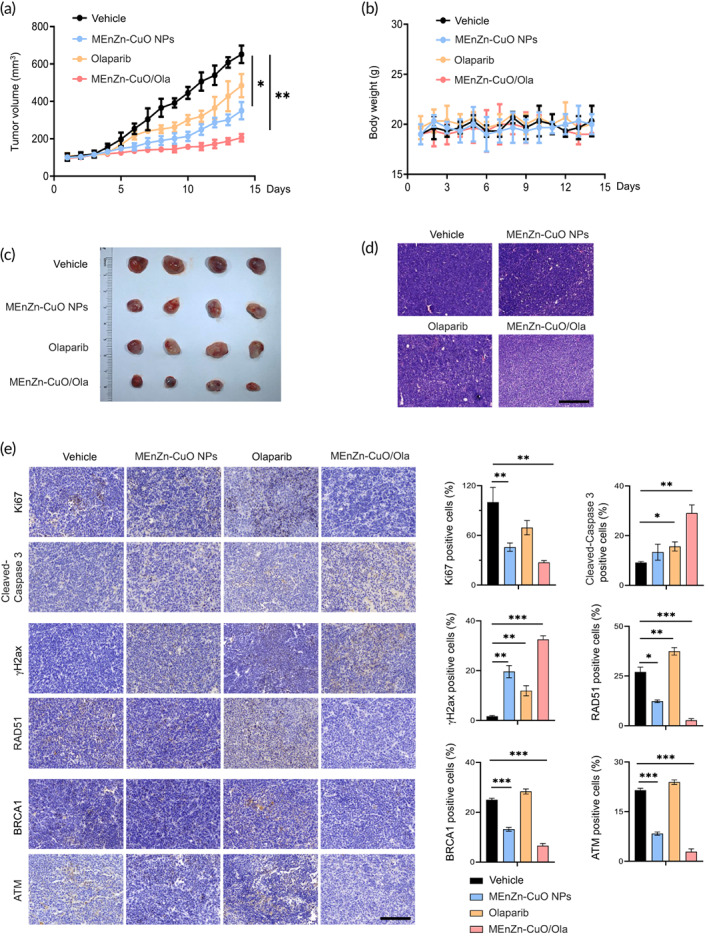
Combined use of Olaparib and MEnZn‐CuO NPs showed synergistic effects in vivo. (a) Tumor growth curves of A2780 xenograft mice treated with Olaparib (50 mg/kg/day) and MEnZn‐CuO NPs (5 mg/kg/day) alone or in combination. Day 0 was the treatment start date. tumor size was measured once daily for 14 days. (b) Body weight change of mice per day during treatment. (c) Pictures of A2780 xenograft tumors isolated from mice in different treatment groups at the end of the experiment. (d) Representative images of HE staining of A2780 xenograft tumors using Olaparib and MEnZn‐CuO NPs alone or in combination for 14 days. Scale bar, 100 μm. (e) Representative images of immunohistochemical staining of A2780 xenograft tumors obtained at the end of treatment. Scale bar, 50 μm. Data are shown as the mean ± SEM. *p < 0.05; **p < 0.01; ***p < 0.001 (one‐way ANOVA with Tukey's multiple comparison test).

Next, we evaluated the in vivo efficacy of the combination of MEnZn‐CuO NPs and Olaparib. The Human A2780 cells were isolated from an untreated patient of ovarian endometroid adenocarcinoma. We used a xenograft mouse model of A2780 cells to assay the activity of the drug combination. The combination treatment did not affect the body weight of the mice, but significantly slowed down the tumor growth (Figure 7a–c). As well, HE staining was performed on the organs of the mice (liver, spleen, lung, and kidney), and the results showed that was no significant damage of the organs (Figure S5A), which indicated that the drug combination was not significantly toxic. Notably, although the combination failed to induce tumor regression, we did observe a significant decrease in the density of viable cells in tumors obtained at the end of treatment in the combination group compared to the single‐drug group (Figure 7d). Furthermore, according to histological analysis, the combination treatment significantly decreased Ki67 (a proliferation marker) but increased Cleaved‐Caspase 3 (an apoptosis marker) staining positive cells (Figure 7e). Meanwhile, the combined use of MEnZn‐CuO NPs and Olaparib resulted in a substantial increase in the formation of γH2AX, along with a significant decrease in RAD51‐positive foci (Figure 7e). Consistent with our in vitro results, we were able to observe a significant downregulation of HR pathway protein (BRCA1 and ATM) in both the MEnZn‐CuO NPs group and the combination group (Figure 7e). The protein results similarly suggested this trend and could induce significant apoptosis in the co‐drug group (Figure S5B). These results corroborate to some extent our hypothesis that MEnZn‐CuO NPs with PARP inhibitors are synthetically lethal by inducing defects in cellular HR repair in ovarian cancer.

## 3 DISCUSSION

Compared to conventional therapies, nanocomposites offer new opportunities for the development of diagnostic and therapeutic tools for cancer and other diseases. In our previous study, we found the Zn‐CuO nanoparticles could inhibit the growth of cancer, but the metal nanoparticle could aggregate in the buffer. Albanese and Chan that compared gold nanoparticle cell uptake of monodispersed nanoparticles with hydrodynamic diameters of 30 to 170 nm to corresponding nanoparticle aggregates.^35^, ^36^ They found that the large aggregation nanoparticles, which are greater than 200 nm, can reduce the uptake of cells. As a result, the MEnZn‐CuO nanoparticles with less than 200 nm may have a more effective uptake in cancer cells compared to the Zn‐CuO nanoparticles with large size than 200 nm. In the present study, we demonstrated that the metal nanoparticles MEnZn‐CuO NPs inhibited ovarian cancer growth both in vivo and vitro. Like Zn‐CuO NPs, MEnZn‐CuO NPs can lead to elevated levels of apoptosis and autophagy. But more importantly, our study showed that MEnZn‐CuO NPs have a role in inducing defective cellular HR repair capacity in ovarian cancer, which allows it to increase the sensitivity of tumors to PARP inhibitors. This result provides preliminary evidence that NPs have the potential to be novel anticancer agents for the treatment of ovarian cancer.

High‐grade serous ovarian carcinoma (HGSOC) is the most common histological subtype of ovarian cancer.^37^ Due to the marked tumor heterogeneity of HGSOC, it remains challenging to model and study this complex disease using appropriate tumor cell line models. In previous studies, A2780 and OVCAR8 were among the conventional tools for ovarian cancer research.^38^, ^39^ However, several rarely used cell lines such as SNU119, OVCAR3 showed a more similar molecular profile to HGSOC patient samples and could serve as the most suitable HGSOC models as reported by Domcke et al.^37^, ^40^ As shown in the Table S1, the HRD status and BRCA mutation status was shown in the table. In the current study, we used different ovarian cancer cell lines to reduce the effect of cell line specificity.

It is well known that the HR pathway plays an important role in the development of ovarian cancer, and a defect in its function would potentially lead to a greater susceptible of tumor cells to drugs that are damaging to the cellular genome, including PARP inhibitors.^14^ Over the years, many metal oxide NPs have been reported to exhibit anticancer activity by inducing autophagy.^41^, ^42^, ^43^ In most cases, elevated cellular autophagy after nanoparticles treatment leads to increased cell death. Zn‐CuO NPs previously developed were shown to induce cellular autophagy in pancreatic cancer to inhibit cell growth exerting antitumor effects,^30^ and our current study shows that MEnZn‐CuO NPs can similarly induce autophagy in ovarian cancer. In most cases, elevated cellular autophagy after nanomaterial treatment is one of the possible causes of cell death.^43^, ^44^ However, there are many nanoparticles that induce protective properties against cancer cells. Silver NPs induce autophagy to promote cell survival, and chemical inhibitors inhibit autophagy to enhance the damage induced by silver NPs in cancer cells.^45^ a recent study by Zhang et al. also confirmed that metal oxide nano‐TIO2 induces autophagy protective effect through antioxidant mechanism.^46^ Therefore, the role of MEnZn‐CuO NPs‐induced autophagy in ovarian cancer deserves further study and judgment.

The HR repair plays an important role in the combination strategy of PARP inhibitors. Mutations in the key HR repair pathway genes BRCA1, BRCA2, and ATM are often critical for PARP inhibitor sensitization.^47^, ^48^, ^49^, ^50^ It has been reported that CDK4/6 inhibitors can induce DNA damage and genomic instability, which in turn can have a synthetic lethal effect with PARP inhibitors. Interestingly, MEnZn‐CuO NPs were found for the first time as metal nanoparticles to negatively regulate the expression of HR repair pathway genes in tumors with defective HR repair capacity. However, our current study has not yet identified the targets of MEnZn‐CuO NPs to regulate the HR repair in tumor cells.^50^ Homologous recombination deficiency (HRD) is the first phenotypically defined predictive marker for therapy with PARP inhibitors in ovarian cancer. A retrospective has shown that HGSOC patients with 68.7% belong to the HRD in China, which was higher than the HRD positive proportion in western countries (50%–60%). In our studies, the MEnZnCuo could synergy with the PARP1 inhibitor to inhibited the ovarian cancer lines. The cell lines were used in the studies included the HRD cell lines (OVCAR3) and non‐HRD cell line (as shown table in the supplement files). In our results, MEnZnCuo have shown promising therapeutic efficacy in vitro and in vivo. In the future, we will further dig deeper into the mechanism of action of MEnZn‐CuO NPs and find the key proteins affecting the combination of MEnZn‐CuO NPs and PARP inhibitors, which will open up a new avenue for the treatment of ovarian cancer.

A large number of early clinical trials of PARP inhibitor‐based combination therapies are currently underway in a variety of human malignancies, including ovarian cancer. The combination of AZD6738 and Olaparib, ZEN003694 combined with Talazoparib, Fluzoparib combined with Bevacizumab, Anlotinib combined With Olaparib, CYH33 in combination with Olaparib, AsiDNATM and so on are recruiting for ovarian cancer trails. However, almost none of these early trials involve the synergistic use with nanomaterials, probably because the preliminary basic experiments are still lacking. And the autophagy was involved in the chemosensitivity of ovarian cancer cells.^51^ Evidences have shown that autophagy plays a critical role in DNA repair, particularly in the process of HR. Previous research has mentioned that LC3 and pUlk1, autophagy proteins, have been found to interact with γ‐H2AX, Rad51, and PARP‐1, which are all involved in maintaining genomic stability.^52^ Another research has also shown that activation of autophagy leads to recruitment of BRCA1/Rad51, which are involved in the HR pathway.^53^ And the ligand YM155 regulates BIRC5, leading to the induction of autophagy that affects genome stability through the downregulation of RAD54L and RAD51, resulting in autophagy‐dependent ROS‐mediated DNA damage.^54^ Our result has shown that MEnZn‐CuO NPs may be an autophagy enhancer to disrupting the autophagy and HR repair to inhibit the ovarian cancer cells by the synergy effect in the Figure S4. The HR and autophagy related HR might involve in synergy effect to inhibit the ovarian cancer. As nano‐sized agents with long circulation times leak into tumor tissue preferentially due to permeable tumor vasculature, then they retained in the tumor bed by their reduced lymphatic drainage.^55^ Some metal nanoparticle shown different physicochemical properties that interacted with the components of tumor environments and reprogramming of tumor environments. Moreover, it is important to note that HR defects occur along a spectrum, which is further complicated by the site‐specific characteristics of tumor environments. Genomic instability fuels inflammatory signaling, which can both suppress and promote tumors. In tumors with genomic instability, immune‐suppressive mechanisms can be therapeutically targeted to inhibit immune checkpoints.^56^ The nanoparticle was effectivity tool to treatment the tumor. In the present study, we demonstrated MEnZn‐CuO NPs as more promising new nanocomposites with high stability and significant anti‐tumor effects. Meanwhile, MEnZn‐CuO NPs can also produce a combined autophagy and lethal effect with PARP inhibitors by inducing HR gene downregulation to inhibited ovarian cancer. Overall, our findings suggest that MEnZn‐CuO NPs may be a promising nanoparticle for cancer treatment, especially beneficial for ovarian cancer patients who are resistant to PARP inhibitors.

## 4 CONCLUSION

The MEnZn‐CuO NPs reported in this study are new metallic nanomaterials with homogeneous nano‐properties, high stability, and remarkable anti‐ovarian cancer effects. In addition to affecting proliferation, migration, apoptosis and autophagy, MEnZn‐CuO NPs can also produce a combined autophagy and lethal effect with PARP inhibitors by inducing HR gene downregulation to inhibited ovarian cancer. Further studies should be conducted to elucidate the targets of action of MEnZn‐CuO NPs and the metabolic of MEnZn‐CuO NPs in vivo. Thus, these findings suggest that MEnZn‐CuO NPs may be novel materials for ovarian cancer treatment.

## 5 MATERIALS AND METHODS

### 5.1 The synthesis and characterization of MEnZn‐CuO NPs

The synthesis and the characterization of the Zn‐CuO was described the previous studies.^57^ Briefly, MEnZn‐CuO NPs were prepared from a mixed solution of copper acetate and zinc acetate at a molar ratio of 3:1, by the sonochemical method. The encapsulation of MEnZn‐CuO NPs was prepared by the next few steps. The mPEG‐PPG micelles in aqueous solution was prepared following the protocols of the previous studies with slight modifications.^58^ In brief, the Zn‐CuO NPs was suspension in ddH2O followed by 4 mm probe sonication for 30 min (200 W, work 5 s and rest 5 s) to disperse the nanoparticles by the Scientz‐IID Ultrasonic Homogenizer (Ningbo Scientz Biotechnology). And then the commercial polyoxypropylene glycol (MW 5000‐6500), PEG6000 and Tween80 was added in the suspension nanoparticle followed by probe sonication for 60 mins (200 W, work 5 s and rest 5 s) to encapsulate the Zn‐CuO NPs and the finial concentration of MEnZn‐CuO NPs was 10 mg/mL at stock solution. The mPEG‐PPG micelles without the Zn‐CuO NPs were used as vehicle. Furthermore, either Zn‐CuO NPs and the large particles was removed by 0.22‐μm filter to remove the big size particles and filter the bacterial. The unique Nanoparticle Tracking Analysis (NTA) were used to determine the size distribution of MEnZn‐CuO NPs by using a NanoSight NS300 with a 405‐nm laser instrument (NanoSight NS300, Malvern Instruments, UK) in ddH2O at room temperature. The characterization of MEnZn‐CuO NPs or Zn‐CuO NPs was identified by using scanning TEM. In brief, 8 μL of MEnZn‐CuO NPs or Zn‐CuO NPs was add on the copper grid. The samples were then stained with 2 wt% uranyl acetate (Zhongjingkeyi Technology, China), and then the images were taken using a JEM‐1400FLASH (JEOL Ltd., Japan).

### 5.2 Cell culture and reagents

IOSE80 human normal ovarian epithelial cell was purchased from iCell Bioscience Inc (China). SKOV3 and A2780 human ovarian cancer cell lines were purchased from iCell Bioscience Inc (China). OVCAR3, OVCAR8, SNU119 and OVSAHO human ovarian cancer cell lines were purchased from YaJi Biological Inc (China). Cells were maintained in culture media (OVSAHO cells in Dulbecco's Modified Eagle Medium; IOSE80, SNU119, A2780, OVCAR8 and OVCAR3 cells in RPMI‐1640 Medium; SKOV3 cells in McCoy's 5A Medium) supplemented with 10% fetal bovine serum and penicillin/streptomycin (100 units/mL) at 37°C and 5% CO_2_. MEnZn‐CuO NPs is a novel doped metal nanomaterial synthesized by our group using the sonochemical method, stock concentration is 10 mg/mL in 4°C. Olaparib (AZD2281) was purchased from Aladdin (USA).

### 5.3 Cell viability assay and determination of drug synergy

Cell viability was assayed using the cell counting kit‐8 assay according to the manufacturer's protocol (Dojindo Molecular Technologies, Japan). Synergistic effects were determined by the Chou‐Talalay method to calculate the combination index (CI).^59^

### 5.4 Wound‐healing assay

Wound‐healing assay was used to evaluate cell migration as described previously.^60^, ^61^ Briefly, cells were seeded in 24‐well plates and grown until confluent state and then cells were scratched using sterile tips. Then the cell monolayer was rinsed twice with PBS to remove debris. Fresh culture medium was added with indicated drugs. The mean width of each scratch was measured using Image Pro Plus (Media Cybernetics) and ImageJ.^62^

### 5.5 Clonogenic assay

Cells were seeded on plates and cultured for 24 h before the initiation of drug treatment. Fresh media containing drugs were replaced every 3 days. At the end point, cells were washed with phosphate buffered solution and subsequently stained with 2% crystal violet for 1 h. Images of stained plates were captured using Molecular Imager (USA). The optical absorbance of bound crystal violet (dissolved in 30% acetic acid) was measured at 595 nm by Multiskan™ FC Microplate Photometer (Thermo Fisher).

### 5.6 Three‐dimensional sphere assay

3D sphere culture experiments were performed as previously described.^63^ Cells were seeded on plates with 50% precoated Matrigel (Corning) plus 50% of medium without serum. Culture medium supplemented with 5% fetal bovine serum and 2% Matrigel was replaced every 3 days. After 48 hours of seeding in the plate, the treatment was performed. After treatment, 3D culture experiments were imaged by inverted phase contrast microscope (Olympus, Japan) and scored according to 3D structure integrity. Over 50 structures were scored for each type of drug treatment.

### 5.7 Western blot analysis

Fresh cells were lysed with RIPA lysis buffer. Protein was separated by SDS‐PAGE and transferred to PVDF membranes. Antibodies against MMP2 (Wanlei,WL03224,1:500), MMP9 (Wanlei, WL03096,1:500), Cleaved‐PARP (Cell Signaling Technology, #5625,1:1000), LC3 (Proteintech,14600‐1‐AP,1:1000), ATG7 (Wanlei, WL02793,1:500), p‐ULK(ser556) (Proteintech, 80218‐1‐RR,1:1000), γH2AX (Cell Signaling Technology, #2577,1:1000), BRCA1 (Beyotime, AF6339,1:1000), BRCA2 (Beyotime, AF6342,1:1000), RAD51(Proteintech,14961‐1‐AP,1:1000), ATM (Wanlei, WL04188,1:500), PARP (Wanlei, WL01932,1:500) and Vinculin (YT4822,1:2000) were used as the primary antibodies. HRP‐conjugated antibodies against mouse or rabbit (1:5000, Proteintech) were used as the secondary antibodies. Immunoblot imaging was performed using the BIO‐RAD ChemiDoc™ XRS+ Molecular Imager. The western blot was normalized to Vinculin.

### 5.8 RNA extraction and RT‐qPCR analysis

Total RNA was isolated by TRIzol reagent (Invitrogen). RNA was synthesized into cDNA using the HiScript II 1st Strand cDNA Synthesis Kit (Vazyme). Taq Pro Universal SYBR qPCR Master Mix (Vazyme) was used to conduct the qPCR analysis. QuantStudio™ Design & Analysis Software was used to analyze the samples. Gene expression was normalized to ACTB. The following primers were used:

ACTB.

5′‐CATGTACGTTGCTATCCAGGC‐3′(Forward).

5′‐CTCCTTAATGTCACGCACGAT‐3′(Reverse).

RAD51.

5′‐GGTCTGGTGGTCTGTGTTGA‐3′(Forward).

5′‐GGTGAAGGAAAGGCCATGTA‐3′(Reverse).

BRCA1.

5′‐GTCCCATCTGTCTGGAGTTGA‐3′(Forward).

5′‐AAAGGACACTGTGAAGGCCC‐3′(Reverse).

BRCA2.

5′‐TGCCTGAAAACCAGATGACTATC‐3′(Forward).

5′‐AGGCCAGCAAACTTCCGTTTA‐3′(Reverse).

ATM

5′‐TTGATCTTGTGCCTTGGCTAC‐3′(Forward).

5′‐TATGGTGTACGTTCCCCATGT‐3′(Reverse).

### 5.9 Flow cytometry analysis

Apoptosis in ovarian cancer cells was analyzed with Annexin V‐FITC Apoptosis Detection Kit (Beyotime, China) according to manufacturer's instructions. Briefly, cultured cells were trypsinized with 0.25% trypsin without EDTA, and then stained with Annexin V‐FITC and Propidium iodide (PI) solution. Stained cells were subjected to flowcytometry analysis on ACEA NovoCyteTM 2070R (ACEA Biosciences).

### 5.10 Immunofluorescence staining analysis

Cells were fixed with 4% formaldehyde in PBS after drug treatment, blocked using 5% BSA, and permeabilized with 0.2% Triton X‐100. The primary antibodies were diluted in 1% BSA and incubated at 4°C overnight. Then, secondary antibodies were added to the samples and incubated at room temperature for 1 hour. Antibodies against RAD51 (Proteintech, 14961‐1‐AP, 1:200) and γH2AX (Cell Signaling Technology, #2577,1:200) were used as the primary antibodies. Antifade Mounting Medium with DAPI was from Beyotime (China). Fluorescent secondary antibodies were used and images were captured with a fluorescence microscope (Olympus, Japan).

### 5.11 Comet assay

A comet assay was performed as previously described.^64^ 100 randomly selected cells were analyzed using Casplab software. The level of DNA damage was presented as percentage of DNA in tail. Images were captured with a fluorescence microscope (Olympus, Japan).

### 5.12 Metaphase chromosome spread assay

Cells were treated with colchicine (0.5 μg/mL) (Beyotime, China) for 12 h prior to harvest. Metaphase spreads were prepared as described previously.^65^ Images were captured with oil lens of microscope (Olympus, Japan).

### 5.13 Autophagy detection by fluorescence microscope

Autophagy flow was analyzed as described previously.^66^ Lipofectamine 3000 (Invitrogen) was used to transfect pBABE‐Puro mCherry‐EGFP‐LC3b(human) (MiaoLing Plasmid Platform, China) into the cells according to the manufacturer's protocol. After transfected for another 24 h, MEnZn‐CuO NPs were added with the appropriate concentration and incubated for 24 h. The autophagy flow was observed under a fluorescence microscope (Olympus, Japan).

### 5.14 Xenograft models and in vivo drug treatment studies

Animal experiments were approved by the Animal Ethics Committee of Southwest Medical University (Permit No. 20210927‐019). Eight‐week‐old female nude mice were purchased from Beijing Vital River Laboratory Animal Technology Co., Ltd. (China) and maintained in a pathogen‐free environment. All animal procedures were conducted under the approval of the Animal Care and Use Committee of Southwest Medical University. Mice were inoculated subcutaneously with A2780 cells. The drug treatment started when the tumor xenografts reached approximately 75 mm^3^. MEnZn‐CuO NPs were dissolved in PBS and administered via oral gavage at 5 mg/kg/day. Olaparib was dissolved in 10% hydroxypropyl‐β‐cyclodextrin for intraperitoneal administration and dosed at 50 mg/kg/day. Tumors and body weight were measured every day. Tumors were calculated using the following formula: tumor volume = (length × width^2^)/2.

### 5.15 Histological and Immunohistochemical staining analysis

Organs and tumors were fixed in 4% buffered formalin overnight before paraffin embedding. Paraffin blocks were sectioned and stained with hematoxylin and eosin. For immunohistochemical staining analysis, Antibodies against Ki67 (Proteintech, 27309‐1‐AP, 1:100), Cleaved‐Caspase‐3 (#9661,1:100), RAD51 (Proteintech, 14961‐1‐AP, 1:200), γH2AX (Cell Signaling Technology, #2577,1:200), ATM (Wanlei, WL04188,1:100) and BRCA1 (Beyotime, AF6339,1:100) were used as the primary antibodies. For each tumor sample, 3‐5 random 40 × fields were scored. Digital images were submitted for quantitative image analysis using Image Pro‐plus software.

### 5.16 Statistical analyses

Differences between two independent groups were calculated using Unpaired Student's *t* test and one‐way ANOVA with Tukey's multiple‐comparisons tests as indicated in the figure legends. *p* values less than 0.05 were considered statistically significant and are denoted as follows: *< 0.05, **< 0.01, and ***< 0.001. All data were analyzed with GraphPad Prism 8 software.

## AUTHOR CONTRIBUTIONS

**Jingyan Yi:** Conceptualization (lead); data curation (lead); funding acquisition (equal); methodology (lead). **Xin Luo:** Data curation (equal); methodology (equal); validation (equal). **Jinshan Xing:** Resources (equal); software (lead); writing – review and editing (equal). **Aharon Gedanken:** Methodology (equal); resources (equal). **Xiukun Lin:** Methodology (equal); validation (equal).

## CONFLICT OF INTEREST STATEMENT

The authors declare that they have no competing interests.

### PEER REVIEW

The peer review history for this article is available at https://www.webofscience.com/api/gateway/wos/peer-review/10.1002/btm2.10507.

## Supporting information


**Figure S1.** (A) The nanoparticle aggregation was capture by the visible of the size of sediment. (B) The retroviral transfection efficiency by the double label LC3 retroviral. (C) The autophagy flux of treatment with vehicle, ZnCuO and MEnZn‐CuO in OVCAR8 and A2780 ovarian cancer cell lines. (D) After MEnZn‐CuO NPs treatment, Western blot was performed to detect the expression of apoptosis, migration, and autophagy‐related proteins in OVCAR8 and A2780 ovarian cancer cell lines. Vinculin was used as a normalization standard.
**Figure S2.** After MEnZn‐CuO NPs treatment, Western blot was performed to detect the DNA damage and HR repair‐related protein expression in OVCAR8 and A2780 ovarian cancer cell lines. Vinculin was used as a normalization standard.
**Figure S3.** (A) The Cell viability of Olaparib combined with MEnZn‐CuO NPs in A2780, OVCAR3, OVCAR8, and SNU119 ovarian cancer cells was measure by CCK8. (B) Ovarian cancer cell lines were cultured in 3D matrigel and drugs‐treated for 10–15 days. Representative pictures were shown. Scale bar, 50 μm.
**Figure** S4. (A) The chromosome aberrations in ovarian cancer cell lines after 48 h treatment with MEnZn‐CuO NPs was detected by metaphase chromosome spread assay. Scale bar, 10 μm. (B) Western blot was performed to detect the expression of apoptosis, DNA damage, and HR repair‐related protein expression in ovarian cancer cell lines. Curve‐shift analysis of combination BRCA1‐IN‐2 with Olaparib in the A2780 (C) and OVCAR3 (D). Curve‐shift analysis of combination rapamycin with Olaparib in the A2780 (E) and OVCAR3 (F). Vinculin was used as a normalization standard. Mean ± S.D. for three independent experiments were shown. *p < 0.05; **p < 0.01; ***p < 0.001 (Student's *t* test).
**Figure S5.** (A) Representative images of HE staining of four principal organs excised from A2780‐tumor‐bearing mice using Olaparib and MEnZn‐CuO NPs alone or in combination for 14 days. Scale bar, 100 μm. (B) Western blot was performed to detect the expression of apoptosis, DNA damage and HR repair‐related protein of the xenografted tumor following treatment with Olaparib and MEnZn‐CuO NPs monotherapy or in combination. Vinculin was used as a normalization standard.
**Table S1.** The HRD status and BRCA mutation status of the cell lines.

## Data Availability

Data available on request from the authors.
