# Supplementary material for: Micelle encapsulation zinc‐doped copper oxide nanocomposites reverse Olaparib resistance in ovarian cancer by disrupting homologous recombination repair
Source: Bioeng Transl Med. 2023 Mar 28;8(3):e10507. doi: 10.1002/btm2.10507 (PMC10189445; doi:10.1002/btm2.10507)

Supplemental Figure S1

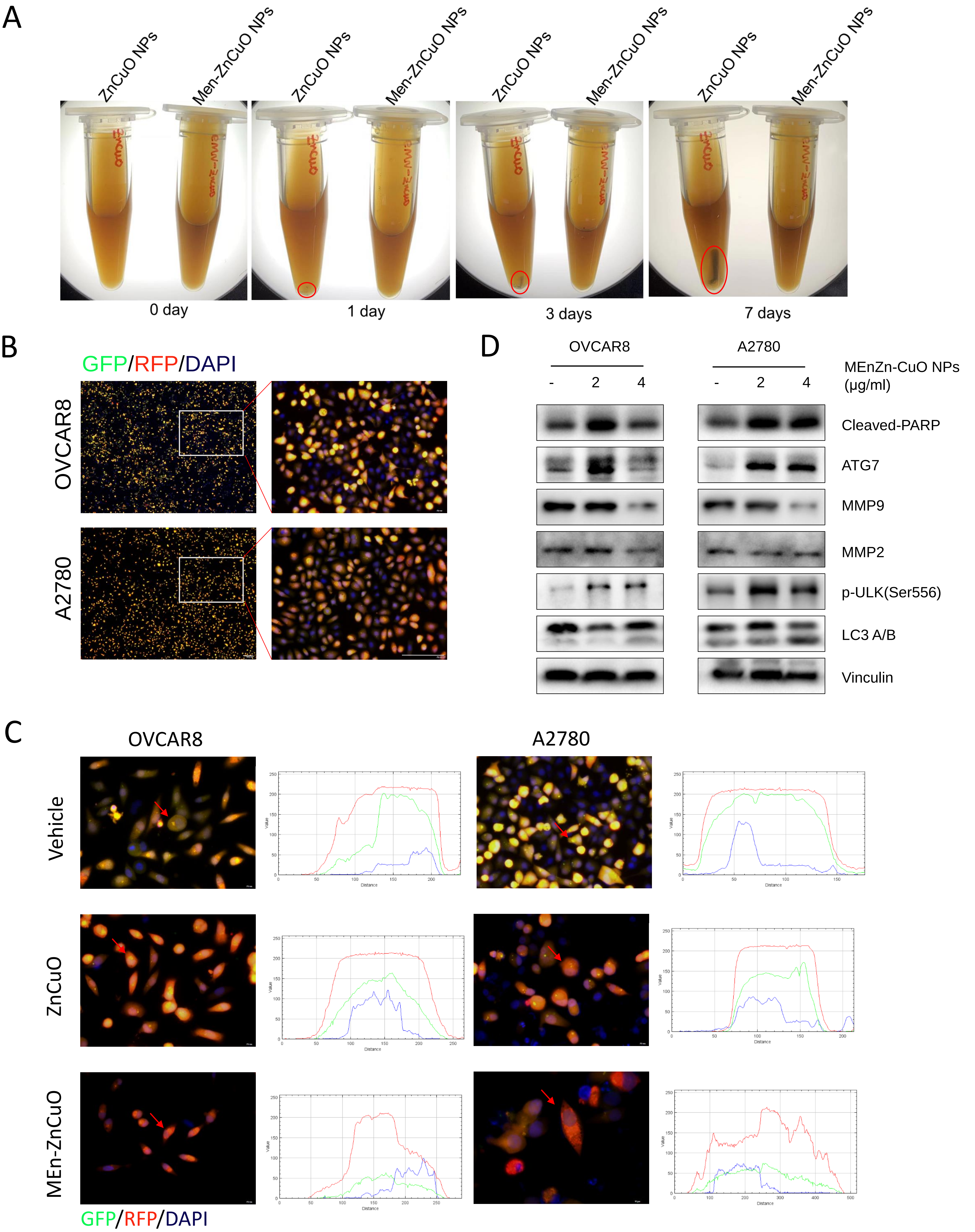

Supplemental Figure S2

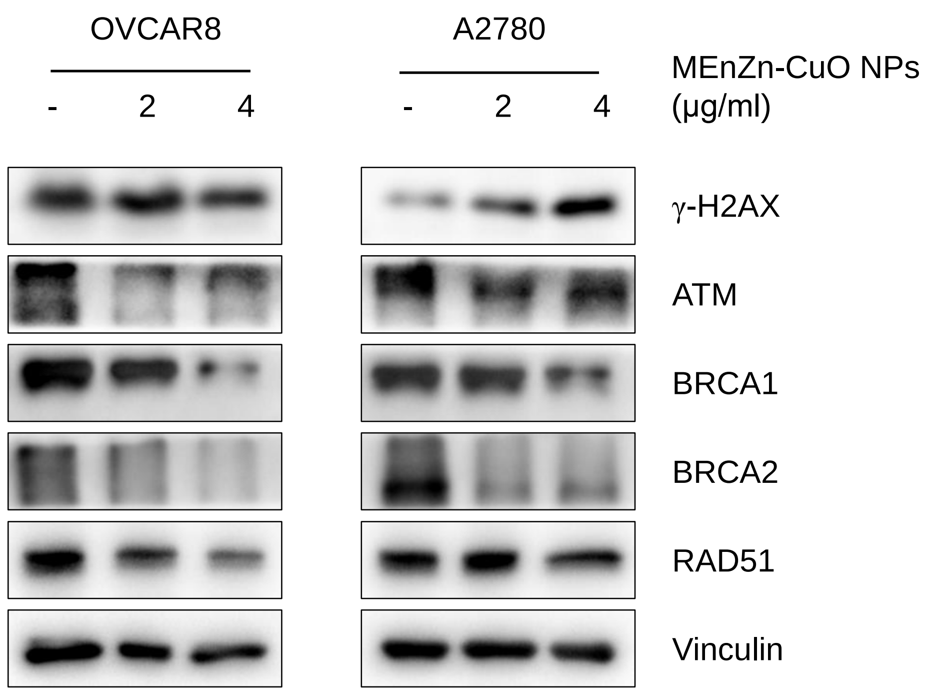

Table 1.The HRD status and BRCA mutation status of the cell lines.

Cell line	OVCAR8	SNU119	A2780	OVCAR3	SKOV3
HRD status	Positive[35]	na	Negative[36]	Positive[36]	Negative[37]
BRCA mutation status [38]	Wild-type	na	Wild-type	Wild-type	Wild-type

na: Not mentioned.

Supplemental Figure S3

A

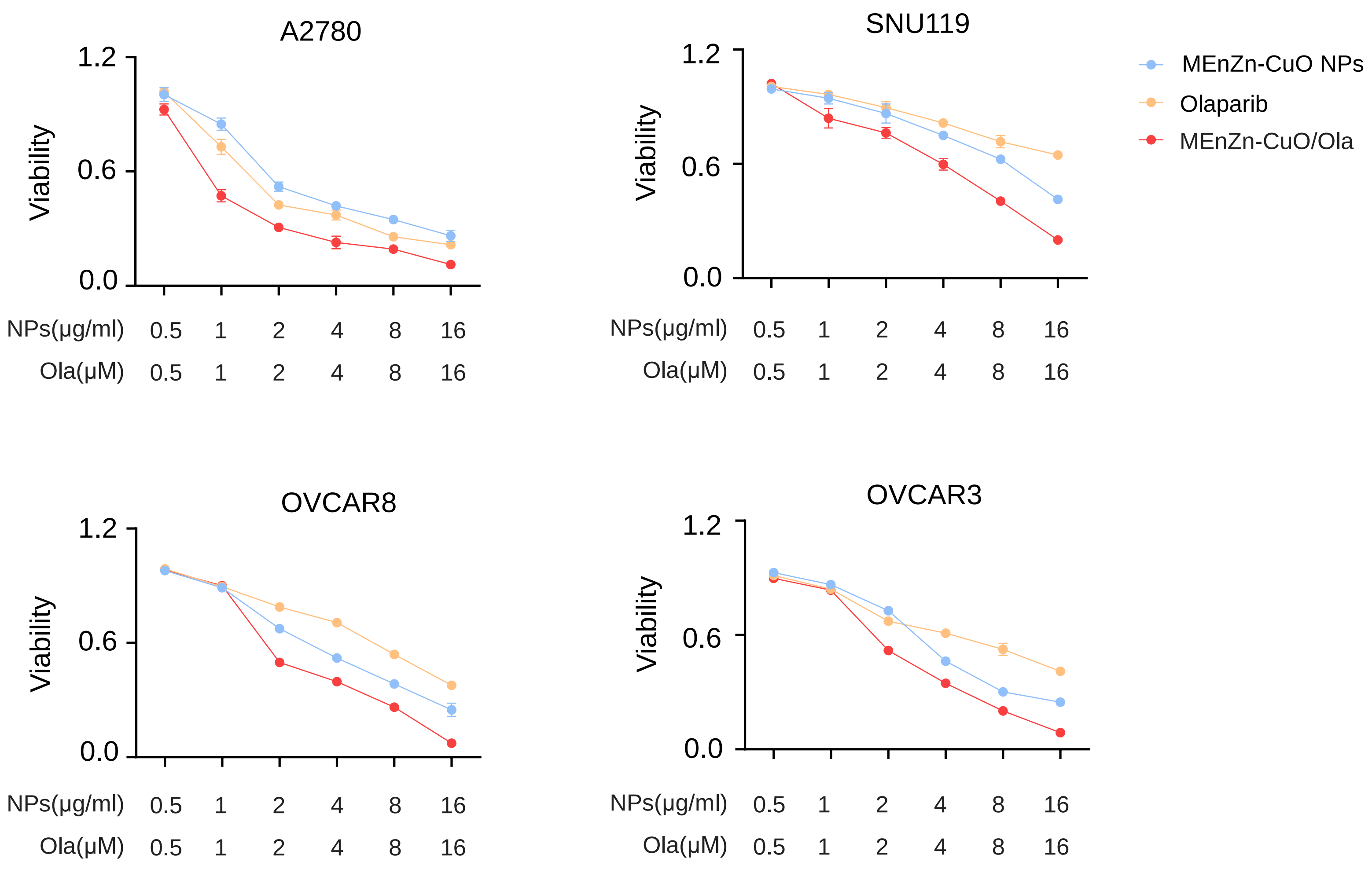

B

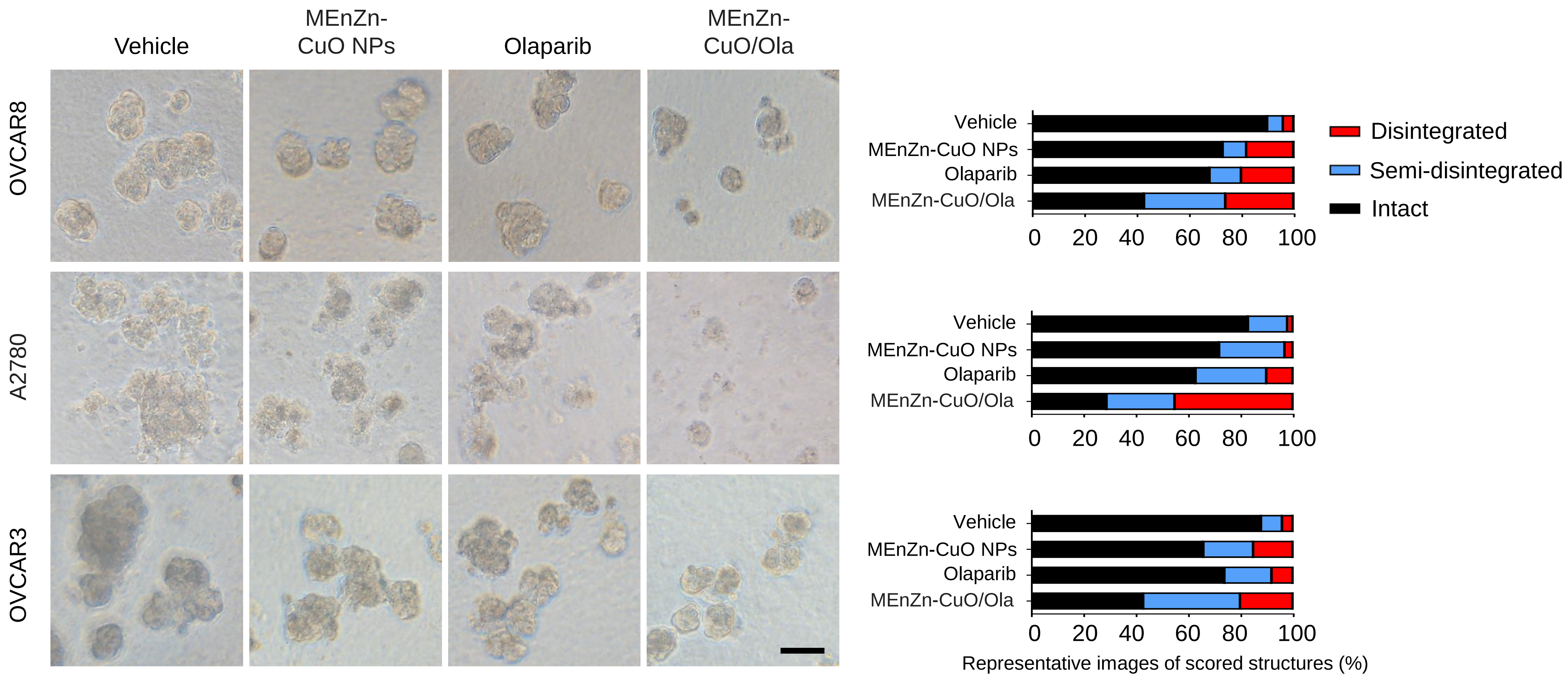

Supplemental Figure S4

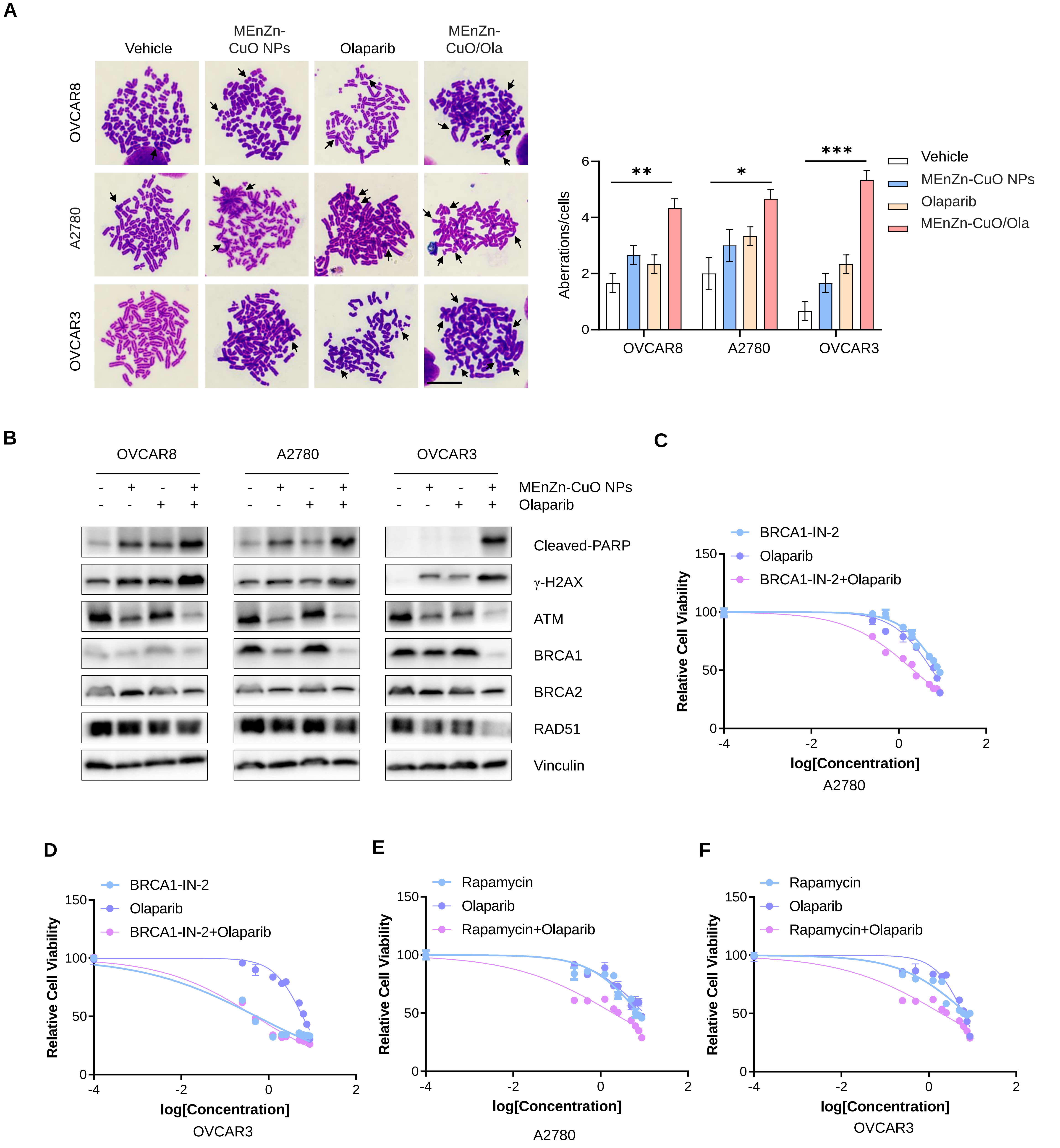

Supplemental Figure S5

A

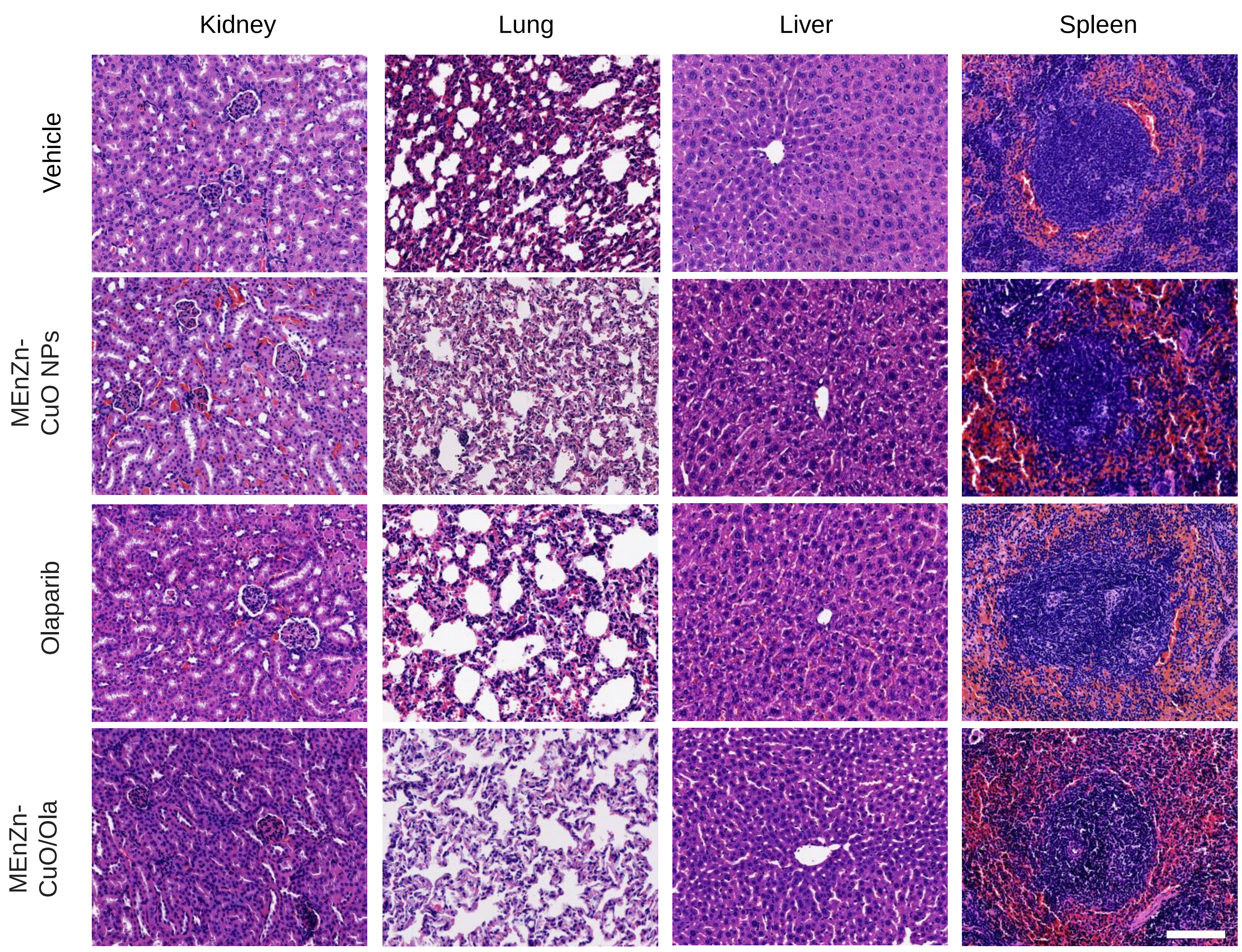

B

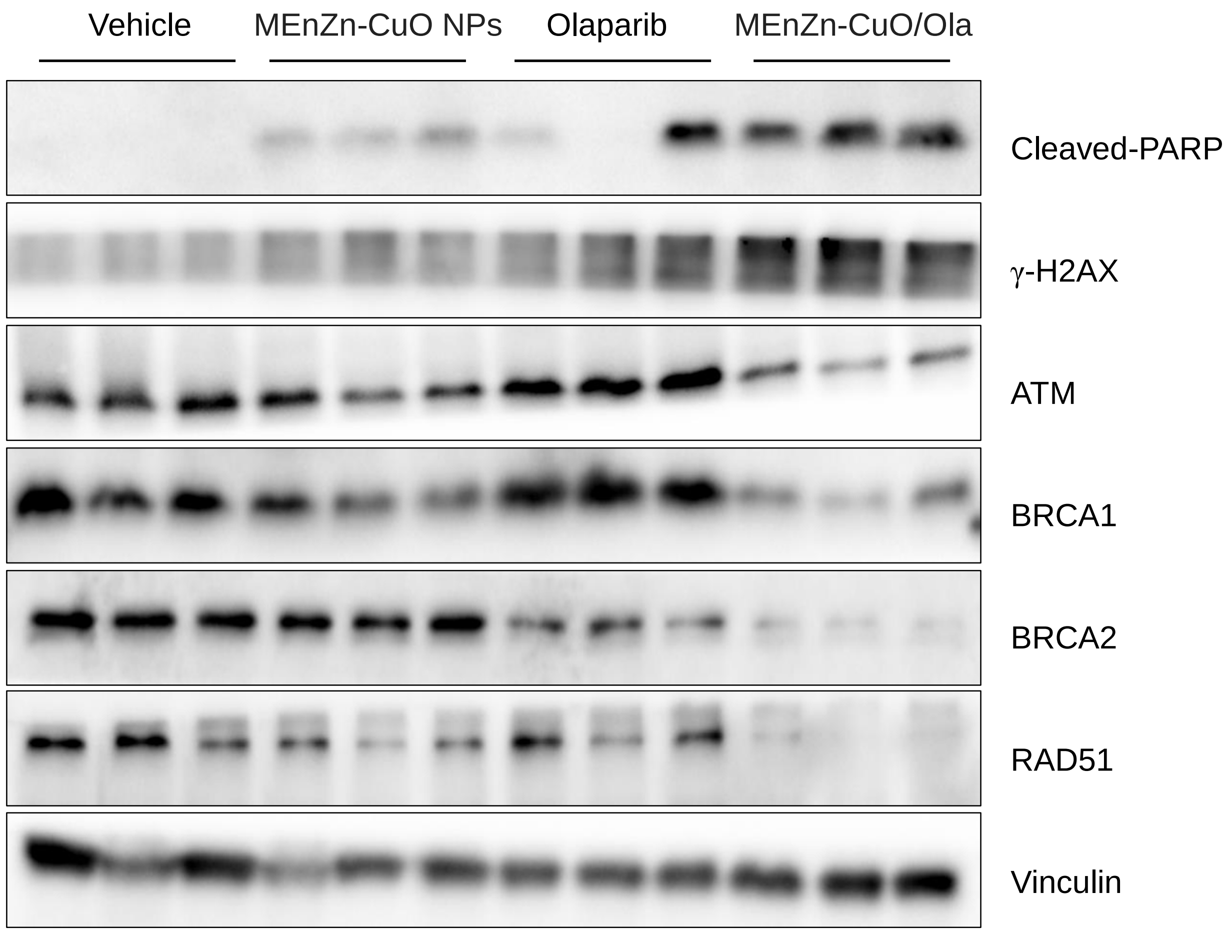

Supplement: Supplementary file 1 — Figure S1. (A) The nanoparticle aggregation was capture by the visible of the size of sediment. (B) The retroviral transfection efficiency by the double label LC3 retroviral. (C) The autophagy flux of treatment with vehicle, ZnCuO and MEnZn‐CuO in OVCAR8 and A2780 ovarian cancer cell lines. (D) After MEnZn‐CuO NPs treatment, Western blot was performed to detect the expression of apoptosis, migration, and autophagy‐related proteins in OVCAR8 and A2780 ovarian cancer cell lines. Vinculin was used as a normalization standard. Figure S2. After MEnZn‐CuO NPs treatment, Western blot was performed to detect the DNA damage and HR repair‐related protein expression in OVCAR8 and A2780 ovarian cancer cell lines. Vinculin was used as a normalization standard. Figure S3. (A) The Cell viability of Olaparib combined with MEnZn‐CuO NPs in A2780, OVCAR3, OVCAR8, and SNU119 ovarian cancer cells was measure by CCK8. (B) Ovarian cancer cell lines were cultured in 3D matrigel and drugs‐treated for 10–15 days. Representative pictures were shown. Scale bar, 50 μm. Figure S4. (A) The chromosome aberrations in ovarian cancer cell lines after 48 h treatment with MEnZn‐CuO NPs was detected by metaphase chromosome spread assay. Scale bar, 10 μm. (B) Western blot was performed to detect the expression of apoptosis, DNA damage, and HR repair‐related protein expression in ovarian cancer cell lines. Curve‐shift analysis of combination BRCA1‐IN‐2 with Olaparib in the A2780 (C) and OVCAR3 (D). Curve‐shift analysis of combination rapamycin with Olaparib in the A2780 (E) and OVCAR3 (F). Vinculin was used as a normalization standard. Mean ± S.D. for three independent experiments were shown. *p < 0.05; **p < 0.01; ***p < 0.001 (Student's t test). Figure S5. (A) Representative images of HE staining of four principal organs excised from A2780‐tumor‐bearing mice using Olaparib and MEnZn‐CuO NPs alone or in combination for 14 days. Scale bar, 100 μm. (B) Western blot was performed to detect the expression o [file BTM2-8-e10507-s001.pdf]
